# Impact of radiomics features, pulmonary emphysema score and muscle mass on the rate of pneumothorax and chest tube insertion in CT-guided lung biopsies

**DOI:** 10.1186/s12931-024-02936-6

**Published:** 2024-08-22

**Authors:** Jakob Leonhardi, Ulrike Dahms, Benedikt Schnarkowski, Manuel Florian Struck, Anne-Kathrin Höhn, Sebastian Krämer, Sebastian Ebel, Gordian Prasse, Armin Frille, Timm Denecke, Hans-Jonas Meyer

**Affiliations:** 1 Department of Diagnostic and Interventional Radiology, University of Leipzig, 49341/9717400 Leipzig, Germany; 2 Department of Anesthesiology and Intensive Care Medicine, University Hospital Leipzig, Leipzig, Germany; 3 Department of Pathology, University Hospital Leipzig, University of Leipzig, Leipzig, Germany; 4 Department of Thoracic Surgery, University Hospital Leipzig, University of Leipzig, Leipzig, Germany; 5 Department of Respiratory Medicine, University Hospital Leipzig, University of Leipzig, Leipzig, Germany

**Keywords:** Computed tomography, Biopsy, Lung cancer, Pneumothorax, Quantitative imaging

## Abstract

Iatrogenic pneumothorax is a relevant complication of computed tomography (CT)-guided percutaneous lung biopsy. The aim of the present study was to analyze the prognostic significance of texture analysis, emphysema score and muscle mass derived from CT-imaging to predict postinterventional pneumothorax after CT-guided lung biopsy. Consecutive patients undergoing CT-guided percutaneous lung biopsy between 2012 and 2021 were analyzed. Multivariate logistic regression analysis included clinical risk factors and CT-imaging features to detect associations with pneumothorax development. Overall, 479 patients (178 females, mean age 65 ± 11.7 years) underwent CT-guided percutaneous lung biopsy of which 180 patients (37.5%) developed pneumothorax including 55 patients (11.5%) requiring chest tube placement. Risk factors associated with pneumothorax were chronic-obstructive pulmonary disease (COPD) (*p* = 0.03), age (*p* = 0.02), total lung capacity (*p* < 0.01) and residual volume (*p* = 0.01) as well as interventional parameters needle length inside the lung (*p* < 0.001), target lesion attached to pleura (*p* = 0.04), and intervention duration (*p* < 0.001). The combined model demonstrated a prediction accuracy of the occurrence of pneumothorax with an AUC of 0.78 [95%CI: 0.70–0.86] with a resulting sensitivity 0.80 and a specificity of 0.66. In conclusion, radiomics features of the target lesion and the lung lobe CT-emphysema score are predictive for the occurrence of pneumothorax and need for chest insertion after CT-guided lung biopsy.

## Introduction

Computed tomography (CT)-guided biopsy of unclear/suspicious pulmonary nodules is routinely used to obtain tissue for histopathology assessment. In comparison with bronchoscopy biopsy, CT-guided biopsies are considered cost effective and yield a higher diagnostic accuracy in regard of the assessment of peripheral located pulmonary lesions or nodules [1–3].

In comparison with surgical approach, percutaneous CT-guided lung biopsies can be considered less invasive and does not require anesthesia [2, 4].

However, CT-guided lung biopsies harbor also interventional complications and risks [3, 4]. Additionally, to the known complications of other CT-guided percutaneous interventions comprising infectious seeding, hematoma and tissue damage, lung biopsies have specific complications, especially the incidence of pneumothorax [3].

Pneumothorax is considered as the most common complication of lung biopsies with yet high and variable reported incidences. According to the literature, the frequency ranges from 9 to 54% throughout the different study populations [5]. A comprehensive analysis of total 9,783 CT-guided lung biopsies reported a frequency of 35% [4]. Notably, the amount of cases with the need of chest tube placement ranged from 2 to 15% of cases [6].

Various studies investigated factors that might influence the amount of postinterventional pneumothorax and consequently therapeutic release with chest tube insertion, including the risk factor pulmonary emphysema [4–10]. Among these, lung emphysema was predominantly assessed visually by the reader, which can be considered subjective and could be influenced by bias.

During recent years, quantitative CT imaging markers are a reliable method to quantify emphysematous lung areas as low attenuation areas in thoracic CT images using a dedicated software tool [5].

To date, only few systematic studies, each only investigating small patient samples, have investigated quantitative imaging methods in these patients. Despite some evidence in favor of quantitative emphysema score as a risk factor for occurrence of pneumothorax [5], some data disapprove it as a risk factor [11].

In the search of better methods to predict pneumothorax in patients at risk following CT-guided lung biopsy, texture analysis is a research field to provide quantitative imaging biomarkers using radiological images [12–15].

Various studies have investigated the clinical benefit of the use of CT texture analysis, most commonly in the research field of oncology [12–15]. Using this approach, texture features can provide imaging information, which provide more insight into microstructure of tissue. It is unclear so far, whether texture analysis could aid to predict pneumothorax risk undergoing CT-guided lung biopsy. In the light of the upcoming increase in low-dose CT studies within the pan-European lung cancer screening programmes for patients at risk of lung cancer, CT-guided lung biopsies will represent an adequate procedure of peripheral lesion in certain patients [16]. The major risk developing pneumothorax for those individuals following transthoracic lung biopsy is mainly caused by a relevant smoking history leading to airway limitation and irreversible parenchymal lung tissue destruction, and thus chronic obstructive pulmonary disease (COPD) and emphysema [17].

The purpose of the present analysis was to elucidate the rate of pneumothorax following CT-guided lung biopsy and to identify risk factors with clinical features and quantitative CT imaging comprising CT lung emphysema score and texture analysis of the target lesion.

## Materials and methods

### Patient acquisition

The present retrospective analysis was approved by the institutional review board (University of Leipzig, register no. 344–2007, Ethics Committee, University of Leipzig, Leipzig, Germany). All patients undergoing CT-guided lung biopsy were retrospectively assessed between the years 2012 and 2021. COPD diagnosis was documented according to patient’s medical history.

### Clinical and respiratory parameters

Comorbidities were extracted from the medical records comprising diabetes mellitus, arterial hypertension and prior malignancy. COPD diagnosis was documented according to patient’s medical history. History of smoking and the amount of pack years (20 cigarettes per pack per day over the years) were extracted from the records.

The COPD was classified accordingly to the Global Initiative for Chronic Obstructive Lung Disease (GOLD) [17]. The pulmonary function test (PFT) measurements included spirometry and body plethysmography and were performed by using the MasterScreen-Body by JAEGER GmbH & Co. KG according to current practice guidelines on technical standards [18, 19] and included forced vital capacity (FVC), forced expiratory volume in 1s (FEV_1_), its ratio (FEV_1_ /FVC), total airway resistance (R_tot_), total lung capacity (TLC), residual volume (RV) and its ratio (RV/TLC) as well as diffusion capacity for carbon monoxide after a single breath (DLCO SB) and DLCO SB referenced to alveolar volume (DLCO/VA) were assessed.

Blood gas analyses (BGA) were performed on capillary blood samples analyzed. BGA included arterial oxygen tension (pO_2_), arterial carbon dioxide tension (pCO_2_) and arterial oxygen saturation (SaO_2_) were performed at the room temperature. PH values, and the alveolar-arterial gradient of oxygen (AaDO2) were calculated [20].

### Procedure of the CT-guided biopsy

The patients gave written informed consent one day before the CT-guided biopsy. The biopsy was only indicated without increased tendency to bleed indicated when platelet count was at least 50.000/mm^3^, partial thromboplastin time ≤ 1.5 times and prothrombin time > 50%. All interventions were performed with the same CT scanner (16-slice CT scanner, Brilliance Big Bore, Philips, Hamburg, Germany). Typical CT parameters were set: 100 kVp; 125 mAs; slice thickness,1 mm; pitch, 0.9).

All biopsies investigated in this study were performed by interventional trained radiologists with at least 2 years of general experience in interventional radiology. The procedure plan, comprising the position and the needle pathway were planned using the last CT images according to clinical routine. Basic principles of the intervention comprised assessing an angle vertical to the parietal pleura, avoiding the lung fissures, large bronchovascular structures, minimizing the pathway of the needle. At last, the passage was planned at the upper part of the ribs to reduce potentially of hematoma [21].

The procedure started with disinfection of the skin and local anesthesia with 10 ml lidocaine 1% (Xylocitin, Jenapharm, Germany) were performed. In all instances, a coaxial 18-gauge biopsy system of a 2 cm throw length needle was used (Bard Mission, Bard Medical, Covington, GA 30014, USA or Biopince, Argon Medical Devices, Athens, TX 75751, USA). During the procedures, CT images were used to validate the accurate localization of the needle tip to reach the target nodule correctly. After removal of the biopsy needle, CT images over the whole lung were acquired to detect post-interventional complications. Patients needed to rest without eating or drinking up to two hours after the procedure to minimize the occurrence of post-biopsy pneumothorax. A chest radiograph was acquired two hours after the biopsy to diagnose complications especially the occurrence of pneumothorax. Patients with pneumothorax on immediate post-biopsy CT images were labelled as “instant pneumothorax”. Cases were classified as “delayed pneumothorax”, when a new pneumothorax was identified on the following radiograph. Cases with a pneumothorax of > 10 mm hem width and/or with clinical symptoms of shortness of breath, raised heart rate and declining oxygen saturation received chest tube insertion.

### CT emphysema score

In the literature, a Hounsfield unit (HU) cut-off value of below − 950 is used to discriminate emphysematous to physiological parenchyma of the lung [5, 22]. For this study, the software package from Philips (Intellispace portal, version 11; Philips, Philips Health System, Hamburg, Germany) was used. Areas of the segmented lung parenchyma − 950 HU were automatically identified and utilized to obtain the total emphysema areas and consecutively provide a score. In short, the total score is calculated as the number of voxels below − 950 HU/voxels of the lung. A description can be found in another study [5]. Moreover, the same measurement was performed for the lung lobe of the target lesion to provide a measurement of the surrounding lung parenchyma.

### Skeletal muscle area

A representative CT-slice at the level of the 12th thoracic vertebral body was used to calculate the skeletal muscle area. The area containing the paravertebral and intercostal muscles was measured on this slice using the freely available software ImageJ 1.48v (National Institutes of Health Image program). A Hounsfield-unit threshold value of -29 to 150 HU was applied to semiautomatically identify the muscle mass area giving the total skeletal muscle area (SMA) of this slice (cm^2^) [23].

### Texture analysis

CT images were further analyzed with the dedicated software MaZda (version 4.7, available at http://www.eletel.p.lodz.pl/mazda/) [24, 25]. A region of interest (ROI) was drawn on the largest slice of the target lesion to calculate the texture features. The ROI was drawn clearly within the pulmonary lesion with two mm distance to the adjacent lung parenchyma. Texture analysis measurements were blindly carried out to the bioptic results by one radiologist (J.L.) with 4 years of general radiological experience. For each ROI, gray-level (µ) normalization was utilized to µ ± 3 standard deviations to reduce the influence of contrast and brightness variation on the texture features, as performed previously [26, 27].

Several different texture features were extracted for each patient including histogram parameters, second order texture features of different groups comprising (co-occurrence matrix run-length matrix, absolute gradient, autoregressive model (theta 1 to 4, sigma), and wavelet transform features. Altogether, 279 texture features were calculated in all lesions.

### Statistical analysis

For this study, statistical analysis was performed with SPSS (IBM, Version 25.0; Armonk, NY, USA). Collected results were primarily assessed with descriptive statistics. Group differences were further analyzed with Mann-Whitney-U test and Fisher’s exact test. Uni- and multivariate logistic regression analysis was carried out to elucidate possible predictors for the occurrence of the postinterventional pneumothorax, given as odds ratio (OR) with 95% confidence interval (CI). Receiver operating characteristics-curve (ROC) with area under the curve (AUC) analyses were used to investigate diagnostic accuracy for the investigated parameters. P-values below 0.05 were used to indicate statistical significance.

## Results

**Fig. 1:**
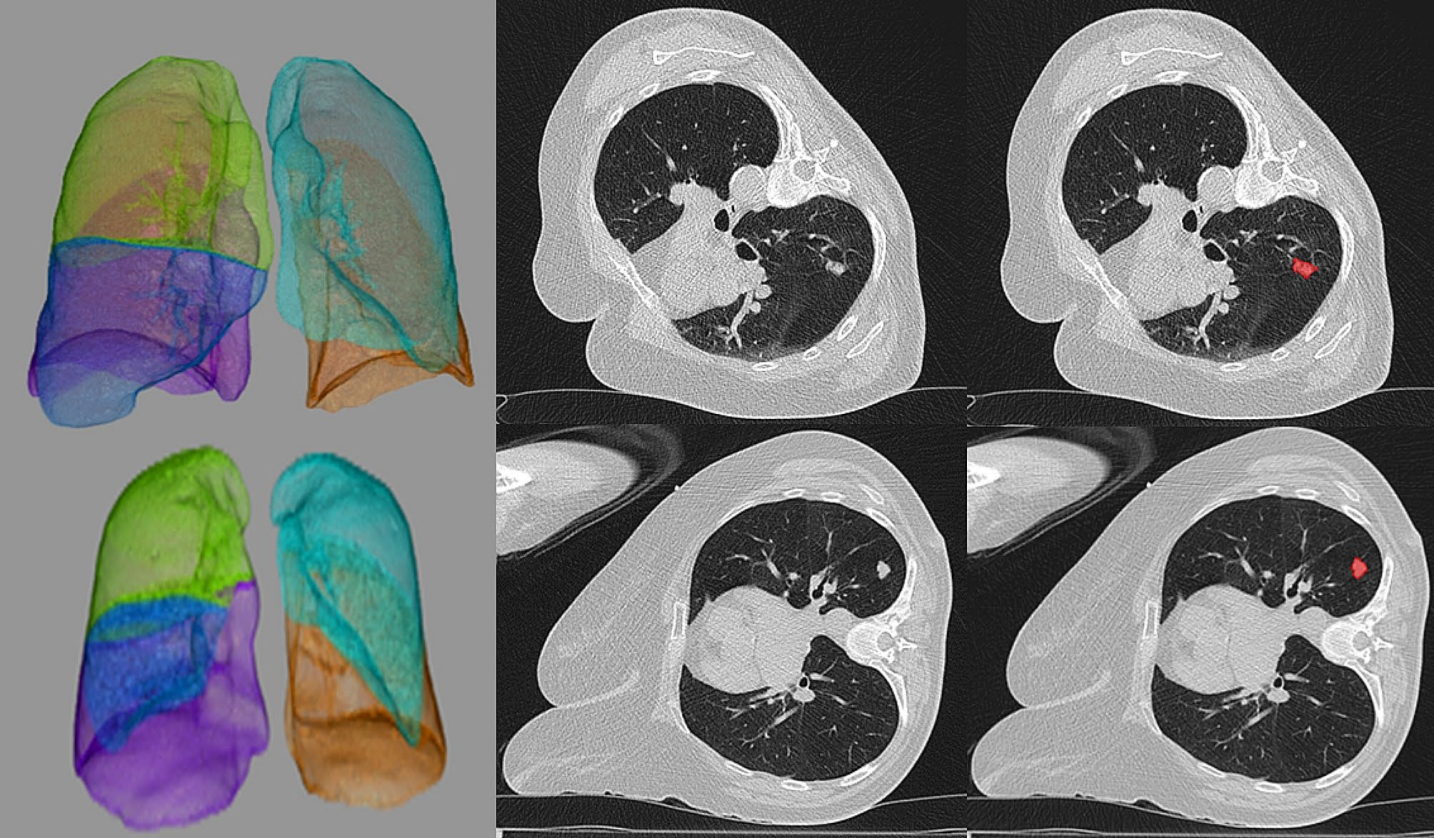
Representative images of the patient sample. First row: segmentation of the lung in order to achieve automated emphysema scoring. Second row: representative slice of the CT Thorax planning scan demonstrating the biopsied lung lesion. Third row: drawn region of the interest (in red) of the biopsied lung lesion. First line: 71-year-old male patient, right-elevated position, no pneumothorax after biopsy, biopsy successful (non-malignant lesion). Second line: 67-year-old female patient, right-lateral position, pneumothorax after biopsy, biopsy successful (metastatic lesion)

Overall, 479 patients (178 female patients, 37.2%) were analyzed. The mean age was 65 ± 11.7 years, ranging from 25 to 91 years. Representative images of the patient sample are displayed in Fig. 1.

### Influences on the occurrence of postinterventional pneumothorax

A total of 180 patients had a postinterventional pneumothorax (37.6%). An overview of the clinical features including group comparisons is given by Table 1.

**Table 1:** Overview of the clinical features of the patient sample

Clinical and puncture feature	No Pneumothorax (*n* = 299)	Any Pneumothorax (*n* = 180)	*p*-value
Gender (female)	119	59	0.12
Age (years)	64.95 ± 12.10	67.56 ± 10.91	**0.02**
Diabetes mellitus	69	42	0.95
Arterial hypertension	170	87	0.07
Prior known malignant disease	161	94	0.73
History of smoking (available in *n* = 478)	248 (82.9%)	158 (88.3%)	0.12
Pack years (available in *n* = 276)	24.11 ± 20.18	23.08 ± 20.58	**0.05**
COPD	46	42	**0.03**
GOLD grade	0.89 ± 1.27	0.88 ± 1.12	0.44
**Lung function testing** (available in *n* = 347 patients)			
FVC in liter (L)	2.72 ± 0.92	2.84 ± 1.01	0.35
FVC in % predicted	77.35 ± 21.64	81.55 ± 22.44	0.09
FEV_1_ in L	2.11 ± 0.88	2.11 ± 0.83	0.80
FEV_1_ in % predicted	74.88 ± 25.89	76.79 ± 24.62	0.40
FEV_1_/FVC	78.15 ± 26.88	74. 44 ± 12.78	0.05
Rtot in % predicted	122.59 ± 86.04	116.89 ± 73.72	0.84
TLC in % predicted	99.04 ± 18.33	105.64 ± 17.70	**< 0.01**
RV in % predicted	133.76 ± 47.11	143.94 ± 43.20	**0.01**
RV/TLC in %	51.46 ± 13.12	52.82 ± 12.39	0.26
DLCO SB in % predicted	57.22 ± 22.44	60.22 ± 20.46	0.36
DLCO/VA in %	71.32 ± 24.28	74.50 ± 21.96	0.31
**Blood gas analysis** (available in *n* = 311 patients**)**			
pO_2_ in mmHg	73.80 ± 12.28	71.95 ± 11.29	0.25
SaO_2_ in %	94.74 ± 2.93	94.40 ± 2.82	0.09
pCO_2_ in mmHg	35.86 ± 4.51	36.41 ± 5.04	0.39
AaDO_2_ in mmHg	28.70 ± 11.21	31.49 ± 10.44	0.89
pH	7.44 ± 0.03	7.44 ± 0.03	0.07
**Biopsy settings**			
Inserted length of the Needle (cm)	3.11 ± 2.08	3.96 ± 2.34	**< 0.001**
Needle angle	62.49 ± 17.53	60.03 ± 18.43	0.15
Lung side (left lung)	123	81	0.41
**Patient positioning**			
Raised on the left	15	10	0.26
Left lateral	41	14	0.89
Raised on the right	22	11	0.96
Right lateral	26	17	**0.03**
Supine position	127	84	**0.002**
Prone position	68	44	**0.01**
Lesion attached to pleura	153	75	**0.04**
Distance to pleura (mm)	0.66 ± 0.89	0.83 ± 1.05	0.09
Lesion solidity (solid)	279	168	0.99
Lesion size (mm)	7.88 ± 13.92	4.58 ± 7.04	**0.04**
Number of biopsies	2.98 ± 3.09	2.66 ± 1.25	0.15
Duration (h: mm: ss)	0:15:21.54 ± 0:08:28.39	0:18:17.55 ± 0:09:17.60	**< 0.001**
Hemorrhage	167	107	0.44
Water lock	36	22	0.95
Diagnostic biopsy	277	149	**< 0.001**

**Abbreviations**:

FVC = Forced vital capacity

FEV_1_ = Forced expiratory volume in 1 s

Rtot in % = Total airway resistance

TLC = Total lung capacity

RV = Residual lung volume

DLCO SB = Diffusion capacity of the lungs for carbon monoxide (single breath)

DLCO/VA = Diffusion capacity of the lungs divided by alveolar volume

pO_2_ = Partial pressure of oxygen

SaO_2_ = Arterial oxygen saturation

pCO_2_ = Partial pressure of carbon dioxide

AaDO_2_ = Alveolar-arterial gradient of oxygen

pH = Potential of hydrogen

Statistically significant differences were identified between the patient groups “no pneumothorax” and “any pneumothorax” for the clinical parameters age (64.95 ± 12.10 vs. 67.56 ± 10.91 years, (OR 1.02; [95% CI 1.00-1.04]; *p* = 0.02), COPD (OR 1.67, [95% CI 1.05–2.67], *p* = 0.03), the total lung capacity (OR 1.02; 95% [CI 0.99–1.05], *p* < 0.01) and the residual lung volume (OR 0.99; 95% [CI 0.98-1.00], *p* = 0.01).

The inserted needle length also showed statistically significant difference between appearance of a pneumothorax or no pneumothorax (3.11 ± 2.08 cm vs. 3.96 ± 2.34 cm; OR 1.19, [95% CI 1.04–1.25], *p* < 0.001). Different ways of positioning the patient showed statistical significance: right lateral (OR 1.09, 95% [CI 0.58–2.08], *p* = 0.03), supine (OR 1.19, [95% CI 0.82–1.72], *p* > 0.01) and prone position (OR 1.10, [95% CI 0.71–1.70], *p* = 0.01) raised the risk of a pneumothorax.

Opposing to that, lesions attachment to the pleura (OR 0.68, [95% CI 0.47–0.99], *p* = 0.04) and greater lesion size (7.88 ± 13.92 mm vs. 4.58 ± 7.04 mm, OR 0.96, [95% CI 0.94–0.99], *p* = 0.04) lowered the risk of pneumothorax. The duration of the intervention differed statistically significant between those without pneumothorax (0:15:21.54 ± 0:08:28.39) and those with pneumothorax (0:18:17.55 ± 0:09:17.60), *p* < 0.001.

Several quantitative CT features were also statistically significant different between the patient groups as provided by Table 2. Total emphysema score was higher in patients with pneumothorax (14.50 ± 11.67 vs. 12.13 ± 10.91, *p* = 0.04, OR 1.02 [95% CI 1.00–1.04]).

**Table 2:** Quantitative CT features between the patient groups with postinterventional pneumothorax and without

Imaging and texture features	No Pneumothorax (*n* = 299)	Any Pneumothorax (*n* = 180)	*p*-value
Skeletal muscle area (cm²)	101.97 ± 25.78	102.62 ± 23.13	0.57
Emphysema score %LAA-950	12.13 ± 10.91	14.50 ± 11.67	**0.04**
Low attenuation volume of biopsied lobe (<-950 HU)	168.42 ± 121.66	213.07 ± 146.87	**0.01**
Total volume of biopsied lobe	890.57 ± 429.71	970.09 ± 404.17	**0.04**
Low attenuation volume ratio in %	17.87 ± 8.75	20.42 ± 8.99	**0.01**
S(1,0)SumAverg	66.11 ± 0.79	66.39 ± 0.68	**< 0.001**
S(1,1)Contrast	80.58 ± 46.22	68.69 ± 35.37	**0.003**
Horzl_RLNonUni	2003.89 ± 2926.31	1152.77 ± 1615.53	**0.003**
Vertl_RLNonUni	2072.66 ± 3015.93	1202.46 ± 1680.25	**0.002**
45dgr_RLNonUni	2172.34 ± 3144.24	1262.45 ± 1741.76	**0.003**
GrMean	2.85 ± 0.74	2.65 ± 0.63	**0.003**
WavEnLL_s-2	14550.74 ± 1528.01	14073.02 ± 1300.99	**< 0.001**

Abbreviations:

HU = Hounsfield units

Also, the low attenuation volume of the biopsied lobe was higher in patients with pneumothorax (168.42 ± 121.66 compared to 213.07 ± 146.87, *p* = 0.01, OR 1.003 [95% CI 1.001–1.004]). Furthermore, the total volume of the biopsied lobe (890.57 ± 429.71 vs. 970.09 ± 404.17, *p* = 0.04, OR 0.99 [95% CI 0.97–1.00]) and the low attenuation volume ratio (17.87 ± 8.75 vs. 20.42 ± 8.99, *p* = 0.01, OR 0.91 [95% CI 0.82–1.00]) were significantly different between the groups.

The texture features “S(1,0)SumAverg” (66.11 ± 0.79 for no pneumothorax vs. 66.39 ± 0.68 for pneumothorax, *p* < 0.001, OR 1.26 [0.75–2.11]) and “GrMean” (2.85 ± 0.74 for no pneumothorax vs. 2.65 ± 0.63 for any pneumothorax, OR 1.02, 0.31–3.33, *p* = 0.03) showed noticeable differences between both categories.

**Fig. 2:**
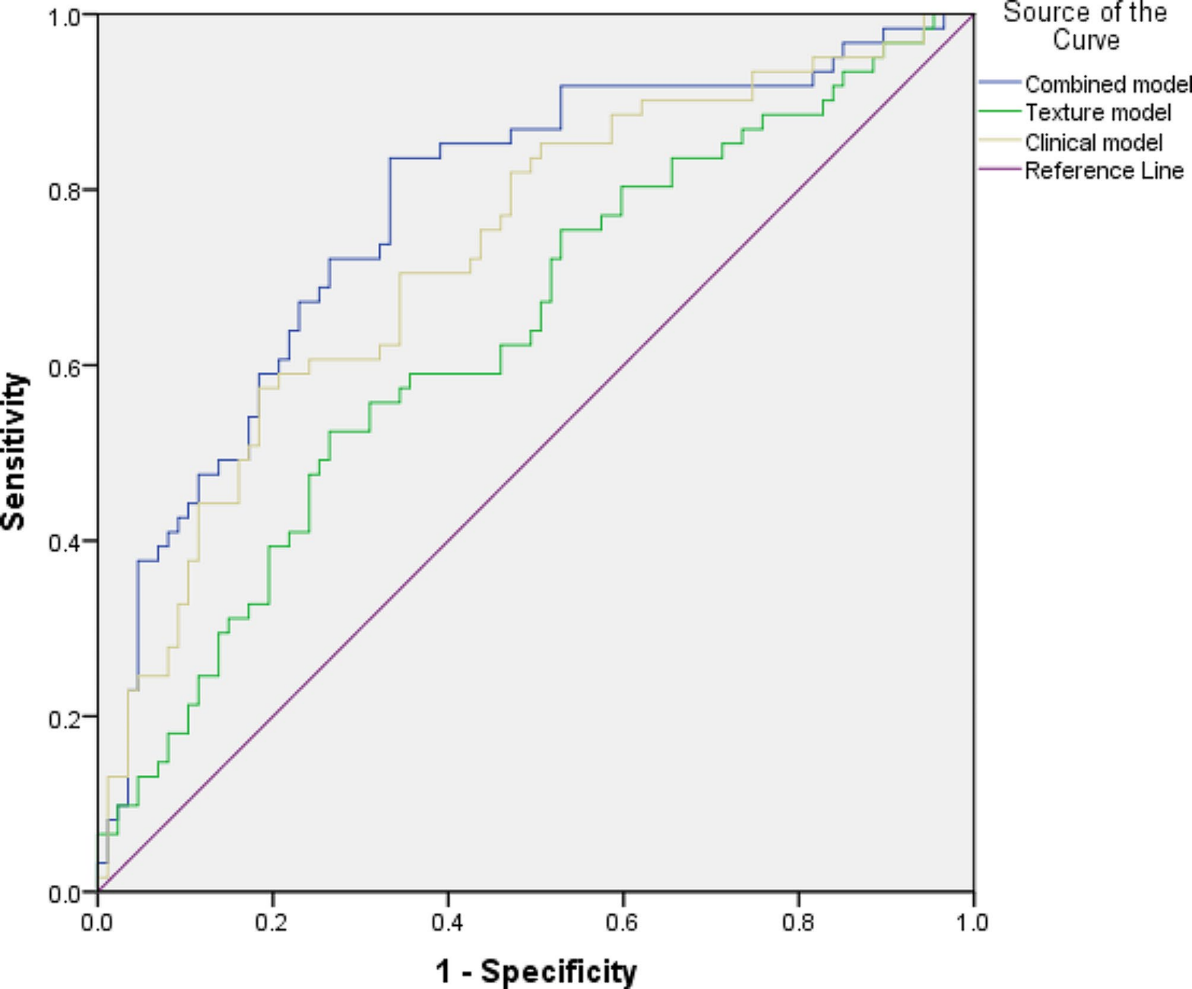
Receiver operating characteristics-curve (ROC) of the prediction of the appearance of a pneumothorax in multivariate logistic regression analysis, clinical model (beige), texture model (green) and combined model (blue). The combined mode achieved an AUC of 0.78 [95%CI: 0.70–0.86], the clinical model achieved an AUC of 0.73 [95% CI: 0.65–0.81], while the texture model an AUC of 0.64 [95% CI: 0.54–0.73]

Multivariate logistic regression was carried out to elucidate the relationships between the statistically significant clinical (*n* = 9), imaging (*n* = 4) and CT texture features (*n* = 7) and the occurrence of any pneumothorax. The combined model demonstrated a prediction accuracy of the occurrence of pneumothorax with an AUC of 0.78 [95%CI: 0.70–0.86] with a resulting sensitivity 0.80 and a specificity of 0.66. Using only clinical features yielded an AUC of 0.73 [95% CI: 0.65–0.81], while the sole texture and imaging feature model resulted in an AUC of 0.64 [95% CI: 0.54–0.73]. The corresponding graphs are displayed in Fig. 2.

### Subgroup analysis of chest tube placement

In 55 of the 180 cases (30.6%) with pneumothorax, there was the need of a chest tube placement after biopsy. The clinical features for these patient groups are shown in Table 3. The statistically significant clinical parameters were the anamnestic presence of COPD (OR 2.86 [95% CI 1.53–5.33], *p* = 0.01), the forced vital capacity (2.62 ± 0.98 for no chest tube necessity vs. 2.96 ± 0.79, OR 1.24 [95% CI 0.76-2.00] *p* = 0.02) and the forced vital capacity in percent (78.47 ± 20.24 for no chest tube necessity vs. 87.32 ± 20.28, OR 1.02 [95% CI 0.99–1.04], *p* = 0.02).

**Table 3:** Clinical features for the patient groups “necessity of chest tube insertion” and “no necessity of chest tube insertion”

Clinical and puncture feature	No necessity of chest tube (*n* = 125)	Necessity of chest tube (*n* = 55)	*p*-value
Gender (female)	44	15	0.28
Age (yrs)	66.94 ± 11.64	69.00 ± 9.10	0.43
Diabetes mellitus	28	14	0.68
Arterial hypertension	58	29	0.46
Prior known malignant disease	67	27	0.61
History of smoking (available in *n* = 179)	106 (85.5%)	45 (81.8%)	0.54
Pack years (available in *n* = 93)	22.60 ± 19.47	23.86 ± 22.59	0.99
COPD	22	20	**0.01**
GOLD stadium	0.90 ± 1.20	0.86 ± 1.09	0.98
**Lung function testing**(available in *n* = 123)			
FVC_L	2.62 ± 0.98	2.96 ± 0.79	**0.02**
FVC in %	78.47 ± 20.24	87.32 ± 20.28	**0.02**
FEV1_L	1.96 ± 0.73	2.17 ± 0.74	0.16
FEV1 in %	74.48 ± 22.49	81.57 ± 26.87	0.16
FEV1/FVC	75.65 ± 13.23	73.22 ± 13.35	0.45
Rtot in %	138.78 ± 97.21	105.75 ± 70.01	0.09
TLC in %	102.95 ± 19.08	105.86 ± 17.79	0.49
RV in %	138.41 ± 45.18	139.75 ± 38.85	0.61
RV/TLC in %	53.56 ± 12.31	49.41 ± 8.76	0.09
DLCO_SB in %	55.64 ± 21.81	58.95 ± 20.93	0.48
DLCO_VA in %	70.37 ± 23.13	70.33 ± 23.12	0.78
**Blood gas analysis**(available in *n* = 111)			
pO_2_ in mmHg	71.50 ± 11.70	74.01 ± 13.69	0.70
SaO_2_in %	94.18 ± 3.31	94.59 ± 2.60	0.73
pCO_2_in mmHg	35.90 ± 4.60	35.70 ± 4.08	0.84
AaDO_2_in mmHg	29.75 ± 10.68	30.83 ± 12.01	0.54
pH	7.44 ± 0.03	7.45 ± 0.03	0.55
**Imaging features**			
Total emphysema score %LAA-950	13.77 ± 10.82	12.90 ± 11.79	0.66
Low attenuation volume of biopsied lobe (<-950 HU)	191.60 ± 125.99	187.41 ± 136.32	0.83
Total volume of biopsied lobe	923.26 ± 406.04	967.50 ± 438.55	0.55
Low attenuation volume ratio in %	19.95 ± 8.39	18.14 ± 9.12	0.30
_MinNorm	55.17 ± 51.88	74.50 ± 55.16	**0.03**
Variance*	1783.12 (1399.96)	1559.98 (1556.57)	**0.04**
Perc,01%	65.92 ± 34.59	79.63 ± 38.98	**0.01**
S(1,0)Contrast*	30.07 (17.76)	35.29 (24.68)	**0.04**
S(1,0)Correlat	0.82 ± 0.08	0.76 ± 0.15	**0.03**
S(1,0)InvDfMom	0.27 ± 0.07	0.24 ± 0.07	**0.05**
S(1,0)DifEntrp*	1.04 (0.13)	1.07 (0.15)	**0.04**
S(1,-1)Contrast*	57.09 (33.63)	67.65 (35.77)	**0.03**
S(1,-1)Correlat	0.64 ± 0.15	0.56 ± 0.21	**0.02**
S(1,-1)DifEntrp*	1.17 (0.14)	1.21 (0.14)	**0.02**
S(4,4)Correlat	0.14 ± 0.17	0.09 ± 0.16	**0.03**
Horzl_ShrtREmp	0.92 ± 0.03	0.93 ± 0.03	**0.05**
45dgr_LngREmph*	1.23 (0.20)	1.19 (0.17)	**0.04**
45dgr_ShrtREmp	0.94 ± 0.03	0.95 ± 0.02	**0.04**
45dgr_Fraction	0.92 ± 0.04	0.93 ± 0.03	**0.05**
Teta1	0.81 ± 0.08	1.4 ± 0.11	**0.04**
WavEnHL_s-1*	107.17 (61.38)	116.29 (96.88)	**0.03**
WavEnHH_s-2*	54.11 (41.62)	70.46 (45.08)	**0.02**
**Biopsy settings**			
Needle length	3.83 ± 2.32	4.32 ± 2.34	0.14
Needle angle	60.35 ± 18.34	59.26 ± 18.93	0.94
Lung side (left lung)	53	28	0.27
**Patient positioning**			
Raised on the left	7	3	0.96
Left lateral	9	5	0.67
Raised on the right	9	2	0.35
Right lateral	14	3	0.28
Supine position	55	29	0.30
Prone position	31	13	0.85
Lesion attached to pleura	56	19	0.22
Distance to pleura (mm)	0.74 ± 0.98	1.03 ± 1.18	0.12
Lesion solidity (solid)	115	53	0.35
Lesion size	4.94 ± 8.12	3.80 ± 3.60	0.47
Amount of biopsies	2.62 ± 1.19	2.76 ± 1.39	0.70
Duration (h:mm:ss)	00:17:26,72 ± 0:08:2.34	00:20:04,02 ± 00:11:32,49	0.34
Hemorrhage	72	35	0.48
Water lock	14	8	0.54
Diagnostic biopsy	103	46	0.82

Values marked with * are reported in mean (interquartile range) because of their skewed distribution.

Abbreviations:

FVC = Forced vital capacity

FEV1 = Forced Expiratory Volume in 1 second

Rtot in % = total airway resistance

TLC = Total lung capacity

RV = Residual lung volume

DLCO_SB = Diffusion capacity of the lungs for carbon monoxide (single breath)

DLCO_VA = Diffusion capacity of the Lungs divided by alveolar volume

pO_2_ = Partial pressure of oxygen

SaO_2_ = Oxygen saturation

pCO_2_ = Partial pressure of carbon dioxide

AaDO_2_ = Alveolar-arterial gradient of oxygen

pH = Potential of hydrogen

HU = Hounsfield units

Regarding quantitative CT features, *n* = 18 parameters were statistically significant different between these groups. The highest significance demonstrated the histogram feature Perc,01% (65.92 ± 34.59 vs. 79.63 ± 38.98, OR 1.01 [95%CI 1.00-1.02], *p* = 0.01) and the second order feature S(1,-1)Correlat (0.64 ± 0.15 vs. 0.56 ± 0.21, OR 0.16 [95%CI 0.02–1.24], *p* = 0.02).

**Fig. 3:**
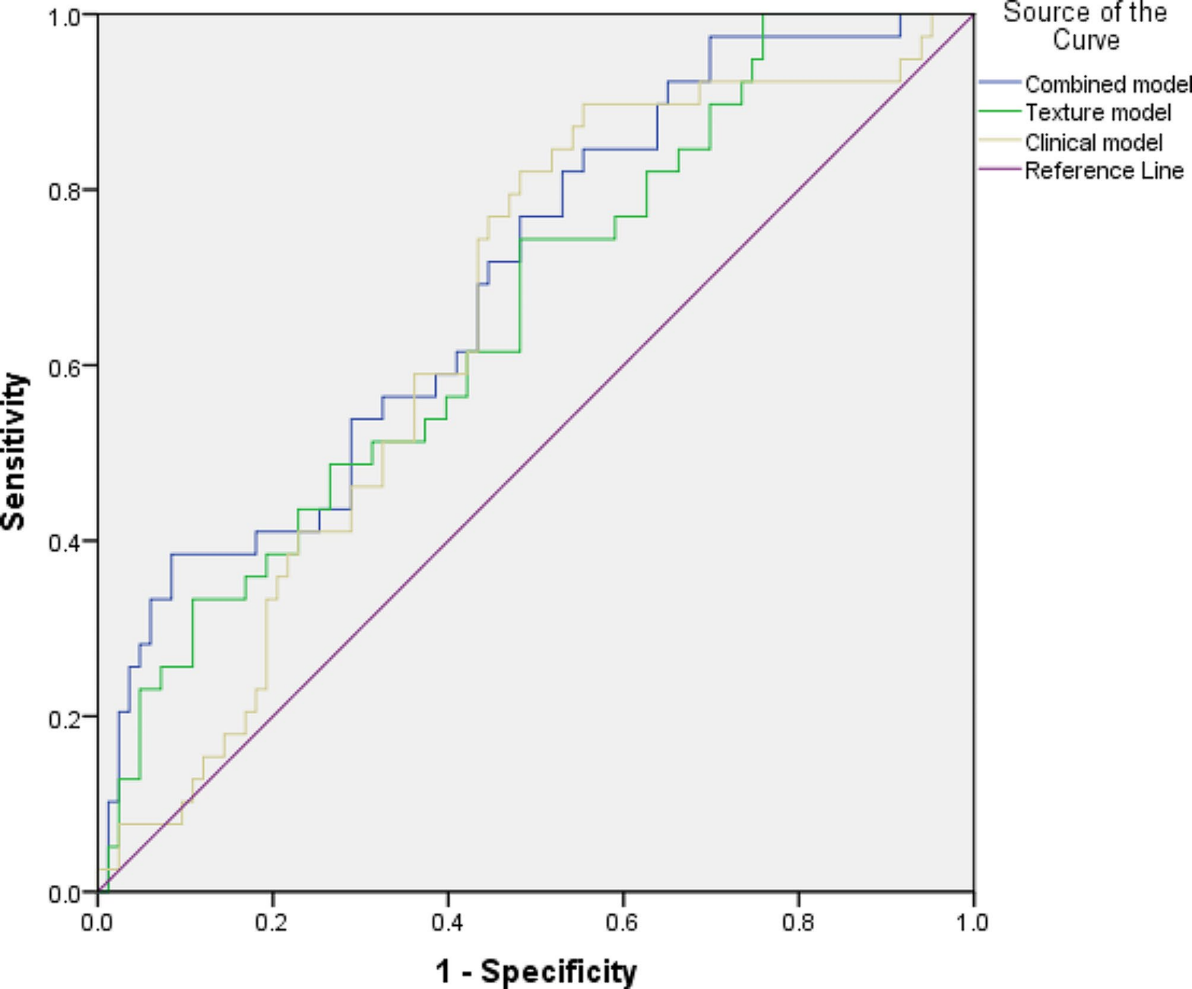
Receiver operating characteristics-curve (ROC) of the prediction of the necessity of a chest tube in the patient subgroup of appearance of any pneumothorax, clinical model (beige), texture model (green), combined model (blue). The combined model achieved an AUC of 0.70 [95% CI: 0.60–0.79], the clinical model achieved an AUC of 0.65 [95% CI: 0.55–0.75], while the texture model achieved an AUC of 0.66 [95% CI: 0.56–0.76]

Multivariate logistic regression was used to analyze the relationship between the significant clinical features (*n* = 3) and the above mentioned highest significant texture features (*n* = 2) with the necessity of a chest tube. The combined model demonstrated a prediction accuracy of the occurrence of pneumothorax with an AUC of 0.70 [95% CI: 0.60–0.79] resulting in an optimized sensitivity of 0.72 with a specificity of 0.55. The sole texture feature model yielded an AUC of 0.66 [95% CI: 0.56–0.76], while the sole clinical model resulted in an AUC of 0.65 [95% CI: 0.55–0.75]. The corresponding graphs are displayed in Fig. 3.

## Discussion

The present study elucidated parameters to predict pneumothorax and the necessity of chest tube insertion after CT-guided lung biopsy by means of clinical features and quantitative CT image parameters based on a large patient sample.

The frequency of pneumothorax following CT-guided lung biopsy reported herein (30.6%) was slightly higher compared to the pooled frequency of 25.3% reported in a meta-analysis comprising 32 studies [3]. However, this might be explained by the fact that only 18G systems were used, which result in a frequency of pneumothorax compared to 20G systems [3].

The investigated CT parameters comprised CT texture analysis, skeletal muscle mass and quantitative emphysema scores. Further factors of the interventional procedure such as characteristics of the punctuated mass and technical aspects were investigated. Several differences were identified between the patient group with the occurrence of pneumothorax such as age, emphysema scores and established diagnosis of COPD in the medical history.

Also, the technical aspect of the inserted length of the needle as well as positioning of the patient showed statistically significant differences. Indeed, right lateral, supine and prone positioning showed significantly higher rates of pneumothorax as did a longer insertion distance of the biopsy needle.

Interestingly, imaging aspects of the punctured lesions also might have played a role in the risk of a pneumothorax: lesion size as well as attachment to pleura and multiple texture parameters were statistically different between both groups highlighting the influence of these parameters on the interventional planning phase. These characteristics are in good agreement with previous analyses [3, 28, 29].

In fact, bigger lesions with less distance to the pleura happened to develop a periinterventional pneumothorax less commonly. This might be caused by smaller and more deeply located lesions being fundamentally easier missed with subsequent need of correction of the needle position and therefore further manipulation putting stress on the pleura. Adding to this, breathing-caused variability of the lesions position relatively to the needle might also be greater in smaller and more deeply located targets. Finally, also duration of the biopsy procedures showed statistical significance, a factor that might similarly be accounted for by more manipulation of the needle and therefore also at the pleura, increasing the risk of changes in the pleural air pressure levels. Skeletal muscle mass and solidity of the punctuated lesions did not differ statistically significant between occurrence of pneumothorax and no pneumothorax.

Regarding emphysema score, our findings are in line with earlier conducted studies who also found these correlations [3, 11, 30, 31]. Two of these studies also found lesion size and inserted needle length to be associated with the risk of a pneumothorax [11, 30]. Also, higher distance to pleura has already been shown to be a risk factor, which is in line with our results. Furthermore, the literature shows that the solidity of punctuated lesions does not cause an increased risk of development of a pneumothorax, which is also matching our results [32]. Interestingly, we also found a statistically significant higher mean age of the patients that developed a pneumothorax when compared to patients without that complication. This finding is somewhat different to the results of studies that have been earlier conducted [33].

In addition, a recent publication stated the “pleura tail sign”, which is a linear extension of the visceral pleura connecting to the lesion, as a possible predictor of a high risk of developing a pneumothorax [34].

However, the impact of CT texture analysis as a risk factor for development of a pneumothorax was not analyzed previously. We identified that numerous texture features showed statistically significant differences in punctuated lesions with peri- or postprocedural development of a pneumothorax when being compared to lesions being punctuated without subsequent pneumothorax. This might be explainable due to the nature of texture parameters as possible biomarkers reflecting lesion heterogeneity [12–15] which has been shown to possibly reflect underlying histopathology features and therefore also the relation between the lung lesion and its surrounding lung parenchyma. When conducting multivariate regression analysis and building a prediction model, a combined clinical and texture feature model even yielded a higher AUC than the sole clinical model. The identified accuracy of the current model is in line to earlier conducted studies that also sought to predict the complication of a pneumothorax in lung biopsies [34].

When looking into the necessity of the insertion of a chest tube, we highlighted the factor of existing COPD being of statistical significance. Moreover, CT texture features describing the heterogeneity of the punctuated masses were also of predictive nature. Interestingly, the emphysema scores did not show any statistically significant differences between the subgroups of pneumothorax with necessity of chest tube and pneumothorax without necessity of a chest tube, which could lead to the conclusion that the emphysema score is only predictive of the occurrence of the pneumothorax but not for the severity.

Only a very limited number of studies sought to examine possible associations of imaging and clinical data with the necessity of chest tube insertion after CT-guided biopsy of lung nodules. Notably, most of them did not evaluate biopsies with an 18G system. Some of these studies found that higher emphysema scores are raising the risk of the necessity of insertion of a chest tube [3, 33, 35], while others like our study did not [31]. A key finding of our study is that the CT-defined emphysema score of the lobe of the target lesion can better predict postinterventional pneumothorax compared to the global CT-emphysema score. It seems plausible that the surrounding lung parenchyma is of greater importance as the biopsy needle passes this parenchyma directly. This new interesting finding should be included in further studies.

One merit of the present analysis was the combination of radiomics features of the target lesion, clinical information and emphysema score to comprehensively predict the postinterventional pneumothorax.

The present analysis is the first investigating the importance of CT-based features of sarcopenia in patients undergoing lung biopsy. Notably, the CT defined muscle mass was not an important risk factor for pneumothorax occurrence, albeit it is a risk factor for various complications throughout surgery and interventional radiology [36–38]. It remains elusive, whether this fact was confounded by the measurement on the level Th12.

In addition, we found many CT-derived texture features of the punctuated lesions to be significantly different between successful biopsies and biopsies without diagnostic result, highlighting this quantitative CT parameters in further analyses.

The present analysis is not free of limitations. First, it is a retrospective study with known inherent bias. Second, although in all cases an 18G needle was used for the biopsy, the needle length varied slightly due to the localization of the target lesion. Third, there is some clinically inconsistency when indicating a chest tube, it is based upon clinical symptoms, diameter of the pneumothorax and even operator’s preference. In some rare cases, another interventionalist might have decided to place a chest tube or not to place it in a different manner. Forth, all clinical parameters were not available for every case, which is caused by the retrospective study design. As CT-guided biopsy patients are a heterogeneous cohort, which are not investigated by pulmonologists in every case.

## Conclusion

Radiomics features of the target lesion and the lung lobe CT-emphysema score are predictive for the occurrence of pneumothorax and need for chest insertion after CT-guided lung biopsy. The identified parameters might aid in clinical decision-making process, and could identify patients at risk for pneumothorax after CT-guided lung biopsy.

## Data Availability

No datasets were generated or analysed during the current study.

## Abbreviations

COPD: Chronic obstructive pulmonary disease
CT: Computed tomography
HU: Hounsfield unit
SMA: Skeletal muscle area
